# Prognosticators and Risk Grouping in Patients with Lung Metastasis from Nasopharyngeal Carcinoma: A more accurate and appropriate assessment of prognosis

**DOI:** 10.1186/1748-717X-6-104

**Published:** 2011-08-26

**Authors:** Xun Cao, Rong-Zhen Luo, Li-Ru He, Yong Li, Wen-Qian Lin, You-Fang Chen, Zhe-Sheng Wen

**Affiliations:** 1 State Key Laboratory of Oncology in South China, Cancer Center, Sun Yat-Sen University, No. 651, Dongfeng Road East, 510060, Guangzhou, China; 2 Department of Thoracic Oncology, Cancer Center, Sun Yat-Sen University, Guangzhou, China; 3 Department of Pathology, Cancer Center, Sun Yat-Sen University, Guangzhou, China; 4 Department of Radiation Oncology, Cancer Center, Sun Yat-Sen University, Guangzhou, China; 5 Department of Anesthesia, Cancer Center, Sun Yat-Sen University, Guangzhou, China

**Keywords:** lung metastasis, nasopharyngeal carcinoma, prognosis, risk subset

## Abstract

**Background:**

Lung metastases arising from nasopharyngeal carcinomas (NPC) have a relatively favourable prognosis. The purpose of this study was to identify the prognostic factors and to establish a risk grouping in patients with lung metastases from NPC.

**Methods:**

A total of 198 patients who developed lung metastases from NPC after primary therapy were retrospectively recruited from January 1982 to December 2000. Univariate and multivariate analyses of clinical variables were performed using Cox proportional hazards regression models. Actuarial survival rates were plotted against time using the Kaplan-Meier method, and log-rank testing was used to compare the differences between the curves.

**Results:**

The median overall survival (OS) period and the lung metastasis survival (LMS) period were 51.5 and 20.9 months, respectively. After univariate and multivariate analyses of the clinical variables, age, T classification, N classification, site of metastases, secondary metastases and disease-free interval (DFI) correlated with OS, whereas age, VCA-IgA titre, number of metastases and secondary metastases were related to LMS. The prognoses of the low- (score 0-1), intermediate- (score 2-3) and high-risk (score 4-8) subsets based on these factors were significantly different. The 3-, 5- and 10-year survival rates of the low-, intermediate- and high-risk subsets, respectively (P < 0.001) were as follows: 77.3%, 60% and 59%; 52.3%, 30% and 27.8%; and 20.5%, 7% and 0%.

**Conclusions:**

In this study, clinical variables provided prognostic indicators of survival in NPC patients with lung metastases. Risk subsets would help in a more accurate assessment of a patient's prognosis in the clinical setting and could facilitate the establishment of patient-tailored medical strategies and supports.

## Background

Nasopharyngeal carcinoma (NPC) is a common epithelial malignancy in southern China [1-3]. The highest incidence has been reported in Guangdong province, where the rate is approximately 20 per 100,000 people per year [1,2]. According to World Health Organisation (WHO) classification based on histological type, most endemic NPCs are type II (non-keratinising carcinoma) and type III (undifferentiated carcinoma), with a high incidence of lymphatic and circulatory metastasis [3,4]. With improvements in the control of local disease due to advanced diagnostic methods, radiotherapeutic techniques and chemotherapy regimens, distant metastasis (DM) is increasingly becoming the major cause of mortality in NPCs [5,6]. The survival period after DM is variable, and long-term survival is improved in patients who receive aggressive multimodality therapy [7-11].

Lung metastasis commonly occurs in NPC [9,12,13]. Some studies have reported that patients with lung metastasis belong to a distinct group with a good prognosis and better survival [8,9,13-15]. Nevertheless, no systematic study has specifically addressed the factors that are associated with lung metastasis in NPC patients. Hence, our retrospective study was designed to examine the relationship between clinical factors and lung metastasis survival (LMS) and overall survival (OS), as well as to identify low-, intermediate- and high-risk subsets that may help in the development of patient-tailored medical support and treatment.

## Methods

### Patients

Subjects were recruited at the Sun-Yat-Sun University Cancer Centre between January 1982 and December 2000. A total of 198 NPC patients with histologically confirmed NPC who were previously untreated, had no evidence of distant metastases (M0) at the time of diagnosis of NPC, received complete response after primary treatment and developed only-lung metastasis(es) at the first failure after primary therapy were eligible for our study. The cases excluded from the current study fulfilled the following criteria: (1) developed extra-pulmonary metastasis at the first failure after primary therapy; (2) did not receive any treatment; (3) did not have adequate clinical information and/or follow-up data. The pre-treatment evaluation included a complete medical history and physical examination, complete blood cell count, serum biochemistry, Epstein-Barr virus (EBV) serology, nasopharyngoscopy, computed tomography (CT) or magnetic resonance image (MRI) scans of the head and neck, chest X-ray and an ultrasound scan of the abdomen. A CT scan of the thorax or the abdomen and a bone scan were performed if the initial examination revealed abnormal findings that were suggestive of metastasis. Forty-five patients had excluded from the present study because the CT chest showed abnormal findings that were suggestive of lung metastasis(es). Clinical stages were assigned according to the American Joint Cancer Committee staging system (AJCC, 1997). The clinical characteristics of the patients are presented in Table 1.

**Table 1:** Patient and disease characteristics of 198 NPC patients with lung metastasis

Characteristics	No. of Patients	%
Gender		
Male	156	78.8
Female	42	21.2
Age (years)		
Median		44.5
Range		20-80
≤45	108	54.5
>45	90	45.5
VCA-IgA		
≤1:320	119	60.1
>1:320	79	39.9
EA-IgA		
≤1:40	128	64.6
>1:40	70	35.4
Histology (WHO)		
Type I	3	1.5
Type II	62	31.3
Type III	133	67.2
AJCC (2002)		
T classification		
T1-T2	83	41.9
T3-T4	115	58.1
N classification		
N0-N1	115	58.1
N2-N3	83	41.9
Overall stage		
I	5	2.5
II	43	21.7
III	108	54.5
IV	42	21.3
Site of metastases		
Unilateral	94	47.5
Bilateral	104	52.5
Number of metastases		
Solitary	65	32.8
Multiple	133	67.2
Size of metastases ^†^		
≤3 cm	142	71.7
>3 cm	56	28.3
Mediastinal nodal metastases ^‡^		
Absent	121	61.1
Present	77	38.9
Locoregional recurrence		
Absent	175	88.4
Present	23	11.6
Secondary metastases		
Absent	149	75.3
Present	49	24.7
DFI (months)		
≤24	108	54.5
>24	90	45.5
Primary treatment		
Radiotherapy	69	34.8
Chemoradiotherapy	129	65.2

Abbreviation: NPC, nasopharyngeal carcinoma; WHO: World Health Organisation; AJCC: American Joint Committee Cancer; DFI: disease-free interval.

^† ^A mass in diameter.

^‡ ^Size in the short-axis diameter.

### Treatment

Radiation therapy was the mainstay of treatment. All patients had planning computerized tomography of the head and neck performed with patient in the treatment position. Computerized tomography-assisted radiation treatment planning was obtained before the initiation of radiotherapy. A 4-MV or 6-MV linear accelerator was used for treatment. The radiation dose ranged from 64 to 70 Gy, according to the tumor stage. Advanced-stage patients (65.2%, *n *= 129) received 4 to 6 cycles of combination chemotherapy (cisplatin/5-fluorouracil) before, during, and/or after radiotherapy. At a clinical examination six weeks later, all patients were in complete remission (CR), as confirmed by endoscopic examination with or without biopsy and a CT or MRI scan of the head and neck.

### Follow-up

After the primary treatment, patients were regularly followed up until death or the last follow-up (follow-up visits occurred every 4-6 months in the first 3 years and every 12 months thereafter). The last follow-up was performed in December 2010. To identify local recurrence or distant metastasis, patients were evaluated with periodic examinations of the nasopharynx. Evaluation of systemic complaints included chest X-rays and abdominal ultrasounds. A CT scan of the chest or abdomen and a bone scan were performed if the initial examination showed abnormal findings that were suggestive of metastasis. If the results of the CT scan were suspicious, lung metastasis was confirmed by biopsy.

Pulmonary metastasis was defined by CT imaging and clinical characteristics on basis of at least two of the following criteria: (1) a soft tissue opacity > 5 mm in the short-axis diameter; (2) peripheral location; (3) multiple lung lesions; (4) patients with advanced stage of the primary NPC; (5) patients with DFI≤ 24 months. These criteria and characteristics have been described and used by some previous literatures and reports[13,16-22].

When lung metastasis(es) was diagnosed, the patient was offered cisplatin-based chemotherapy. Fifty-seven cases (chemotherapy group) received palliative resection or radiotherapy in addition to chemotherapy. One hundred and forty-one patients (chemoradiotherapy group), especially the patients with multiple lung metastases (*n *= 133), received only chemotherapy. The treatment distribution of patient with solitary lung metastasis were 32 chemotherapy-only patients, 12 chemoradiotherapy patients and 21 chemotherapy plus palliative resection patients. The treatment distribution of patients with multiple lung metastases were 109 chemotherapy-only patients, 17 chemoradiotherapy patients and 7 chemotherapy plus palliative resection. The patients with local recurrence received a second course of external radiotherapy (*n *= 23).

The survival status was verified using the best available methods, including verifying the clinical attendance records and with direct telecommunication with the patient or their family.

### Statistical Analysis

Disease-free interval (DFI) was defined as the interval between the onset of the primary treatment and the time of the first diagnosis of lung metastasis(es). Overall survival (OS) was defined as the time from the date of primary treatment to the date of death or the final clinical follow-up. Lung metastatic survival (LMS) was defined as the interval between the date of first diagnosis of lung metastasis(es) and the date of death or final follow-up. The factor analysis for OS and LMS included gender, age, VCA-IgA titre, EA-IgA titre, T classification, N classification, site of metastases (location of pulmonary metastasis, unilateral or bilateral), number of metastases, size of metastases, mediastinal nodal metastases, local recurrence, secondary metastases [subsequent metastases, any distant organ metastasis(es) just presented after lung metastasis(es)], and DFI. The actuarial OS and LMS were estimated using the Kaplan-Meier method, and the differences between the survival curves were compared using the log-rank test. The Cox proportional hazards regression model was used to assess the prognostic significance of the different factors. Statistical significance was defined as *P *< 0.05. The statistical analyses were performed using the SPSS 13.0 software package (SPSS, Inc., Chicago, IL).

## Results

### Patients and Disease Characteristics

A total of 198 patients (156 male and 42 female) were included in this study. The median age was 44.5 years (range, 20 to 80 years). Increased titres of VCA-IgA and EA-IgA were detected in 39.9% (*n *= 79) and 35.4% (*n *= 70) patients, respectively. The histological types of 98.5% of the patients were non-keratinising or undifferentiated carcinoma (WHO type II or III). The distribution of patients within the T classifications were 83 T1-T2 patients (41.9%) and 115 T3-T4 patients (58.1%). The distributions in the N classifications were 115 N0-N1 patients (58.1%) and 83 N2-N3 patients (41.9%). Approximately half of the patients had bilateral metastases (52.5%). DFI≤ 24 months occurred in 108 patients (54.5%), compared with DFI > 24 months in 90 patients (45.5%). Most cases had lung metastasis(es) without local recurrence (88.4%) and/or secondary metastases (75.3%). In total, 133 patients (67.2%) had multiple lung metastases, and 61.1% (*n *= 121) of those patients did not have mediastinal node metastases. Metastases ≥3 cm in diameter was present in 56 cases (28.3%). The details are listed in Table 1.

### Survival Analysis

All the patients were followed up regularly and the last follow-up was carried out in December 2010, 143 cases developed cancer-related deaths (lung metastasis or secondary metastasis).

**Figure 1:**
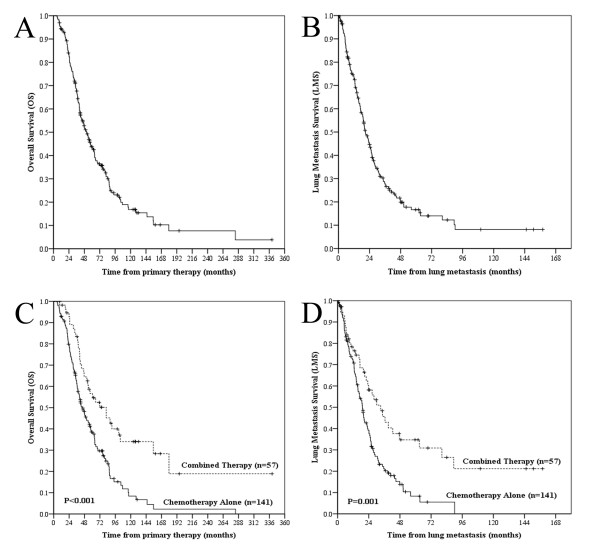
**Kaplan-Meier survival analysis according to different groups**. Overall survival (OS) (A) and lung metastasis survival (LMS) (B) for the entire cohort. Comparison of overall survival (C) and lung metastasis survival (D) between patients treated with combined therapy and chemotherapy alone.

The median OS and LMS for the entire cohort were 51.5 months (range, 5.4 to 340.2 months) and 20.9 months (range, 0.3 to 157.9 months), respectively (Figures. 1A and 1B). Median OS was 37.7 months longer in chemoradiotherapy group (81.8 months) than in the chemotherapy-only group (33.1 months) (*P *< 0.001) (Figures 1C). Median LMS was also longer in chemoradiotherapy group than in the chemotherapy-only group (44.1 months *vs. *19.1 months, *P *= 0.001) (Figure 1D). More than half (54.5%, *n *= 108) of the subjects developed lung metastasis(es) within the first 2 years after primary treatment. After adjustment for clinicopathological characteristics, the modality was still statistically significant for the OS and the LMS *(P *= 0.001, *P *= 0.002, respectively).

### Univariate Analysis of Clinical Variables

Several factors (age > 45 years, VCA-IgA titre > 1:320, bilateral lung metastases, multiple lung metastases and secondary metastases) were significantly related to shorter LMS in the univariate analysis. Moreover, variables that were statistically significant negative predicators of OS included age > 45 years, AJCC T3-T4 classification, AJCC N2-N3 classification, bilateral lung metastases, multiple lung metastases, secondary metastases, and DFI≤24 months (Table 2).

**Table 2:** Univariate analysis of clinical variables for LMS and OS

	LMS			OS		
	
Clinical Variable	HR	95%CI	*P *value *	HR	95%CI	*P *value *
Gender	1.084	0.739 to 1.591	0.681	1.645	0.726 to 1.563	0.747
Age	1.579	1.132 to 2.202	0.007	1.731	1.241 to 2.414	0.001
VCA-IgA (≤1:320 *vs. *>1:320)	1.595	1.067 to 2.383	0.022	1.358	0.909 to 2.028	0.135
EA-IgA (≤1:40 *vs. *>1:40)	1.038	0.687 to 1.566	0.861	1.153	0.762 to 1.743	0.501
AJCC T classification	1.316	0.939 to 1.845	0.110	1.610	1.139 to 2.276	0.007
AJCC N classification	1.355	0.972 to 1.889	0.073	1.469	1.050 to 2.056	0.024
Site of metastases ^1^	1.576	1.127 to 2.205	0.008	2.017	1.433 to 2.840	<0.001
Number of metastases ^2^	1.669	1.155 to 2.413	0.006	2.042	1.404 to 2.971	<0.001
Size of metastases ^3 †^	1.034	0.710 to 1.504	0.863	1.428	0.981 to 2.079	0.063
Mediastinal node metastases ^4 ‡^	1.061	0.753 to 1.496	0.735	1.234	0.875 to 1.740	0.230
Locoregional recurrence ^5^	1.277	0.787 to 2.071	0.323	1.058	0.650 to 1.719	0.822
Secondary metastases ^6^	3.100	2.116 to 4.541	<0.001	1.830	1.263 to 2.652	0.001
DFI (months, ≤24 *vs*. >24)	1.330	0.950 to 1.860	0.096	4.209	2.923 to 6.060	<0.001

Abbreviation: LMS: lung metastasis survival; OS: overall survival; AJCC: American Joint Committee Cancer; DFI: disease-free interval; HR: hazard ratio; 95%CI: 95% confidence interval.

^1 ^Unilateral *vs. *Bilateral; ^2 ^Solitary *vs. *Multiple; ^3 ^≤ 3 cm *vs. *> 3 cm; ^4 ^Absent *vs. *Present; ^5 ^Absent *vs. *Present; ^6 ^Absent *vs. *Present.

^† ^A mass in diameter.

^‡ ^Size in the short-axis diameter.

* Cox proportional hazards regression models.

### Multivariate Analysis of Clinical Variables

**Figure 2:**
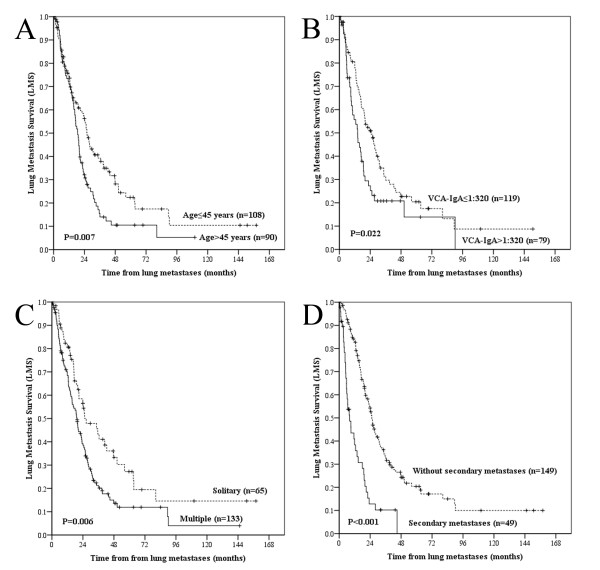
**Lung metastasis survival curves according to age, VCA-IgA titre, number of metastases and secondary metastases**. Comparison of lung metastasis survival (LMS) according to age (A), VCA-IgA titre (B), number of metastases (C), and secondary metastases (D).

**Figure 3:**
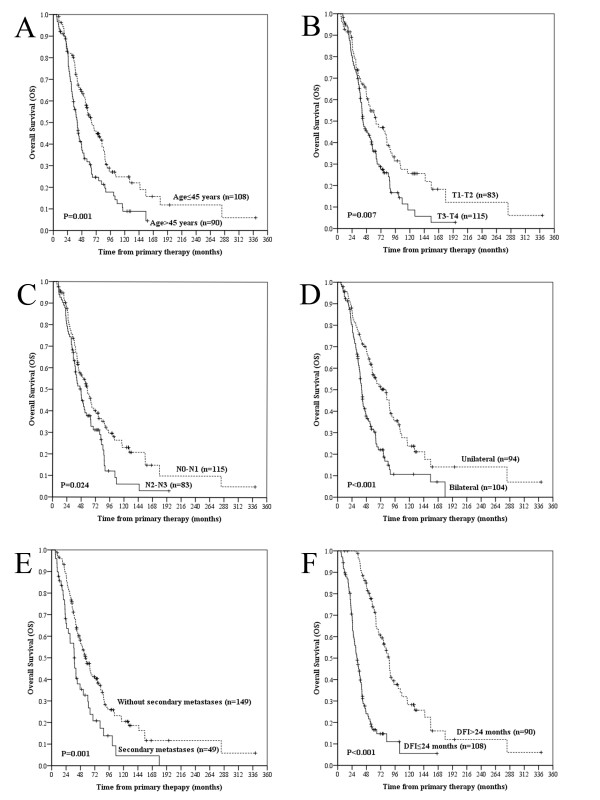
**Overall survival curves according to age, T classification, N classification, site of metastases, secondary metastasis and disease-free interval**. Comparison of overall survival (OS) according to age (A), T classification (B), N classification (C), site of metastases (D), secondary metastasis (E), and disease-free interval (DFI) (F).

In the multivariate analysis of the clinical variables for LMS, all of the univariate variables were independently significant predictors (Figure 2) with the exception of the site of metastases. Independently negative prognostic factors for OS included age > 45 years, AJCC T3-T4 classification, AJCC N2-N3 classification, bilateral lung metastases, secondary metastases, and DFI≤24 months (Figure 3). The hazard ratios (HR), the 95% confidence intervals (CI) and the *P *values are presented in Table 3.

**Table 3:** Multivariate analysis of clinical variables for LMS and OS

Clinical				
endpoint	Variable	HR	95% CI	*P *value *
LMS	Age	1.659	1.107 to 2.484	0.014
	VCA-IgA	1.518	1.012 to 2.277	0.043
	Site of metastases ^1^	1.033	0.606 to 1.757	0.906
	Number of metastases ^2^	1.585	1.013 to 2.481	0.044
	Secondary metastases ^3^	3.132	1.948 to 5.036	<0.001
OS	Age	1.906	1.355 to 2.682	<0.001
	AJCC T classification	1.530	1.074 to 2.177	0.018
	AJCC N classification	1.622	1.149 to 2.289	0.006
	Site of metastases ^1^	1.464	1.023 to 2.095	0.037
	Number of metastases ^2^	1.079	0.630 to 1.848	0.782
	Secondary metastases ^3^	2.343	1.585 to 3.462	<0.001
	DFI	5.050	3.356 to 7.576	<0.001

Abbreviation: LMS: lung metastasis survival; OS: overall survival; AJCC: American Joint Committee Cancer; DFI: disease-free interval; HR: hazard ratio; 95% CI: 95% confidence interval.

^1 ^Unilateral *vs. *Bilateral; ^2 ^Solitary *vs. *Multiple; ^3 ^Absent *vs. *Present

* Cox proportional hazards regression models.

### Identification of Low-, Intermediate-, and High-risk Subsets

Based on the univariate and multivariate analyses of the clinical variables, we were able to classify the 198 cases into three subsets according to the presence of independently significant, negative prognostic factors (age, VCA-IgA, T classification, N classification, site of metastases, secondary metastases, number of metastases and DFI) for survival.

**Figure 4:**
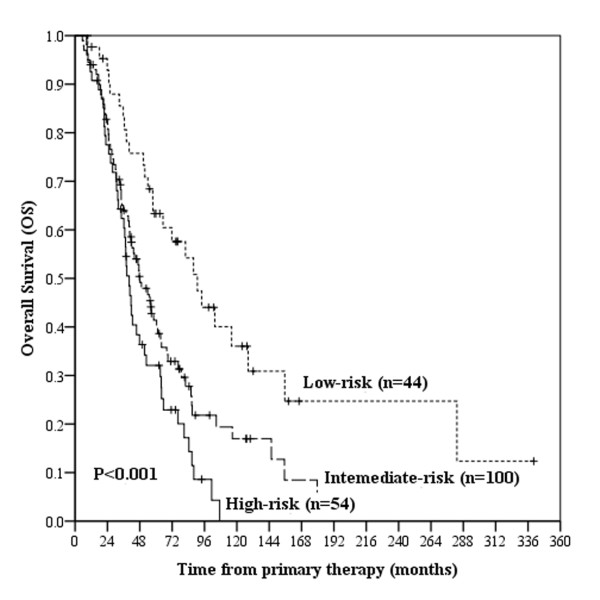
**Kaplan-Meier survival analysis according to different risk subset**. Comparison of overall survival (OS) among the low-risk subset, the intermediate-risk subset and the high-risk subset.

A score of 1 was provided if an independently significant negative prognostic factor was present. A score of 0 was assigned if the prognostic factor was absent. Scores were totalled for each patient, and the patients were then subdivided into three risk subsets. The low-risk subset included the patients with 0-2 independent prognostic factors (score 0-2), the intermediate-risk subset included the patients with 3-4 independent prognostic factors (score 3-4) and the cases who had more than 4 independently significant negative factors were classified into the high-risk subset (score 5-8). There were 44, 100 and 54 patients in the low-, intermediate- and high-risk subsets, respectively (Table 4). The median survival periods for those three subsets were 90.7, 48.2 and 40.2 months, respectively (*P *< 0.001). The survival curves stratified by risk subset are shown in Figure 4.

**Table 4:** Identification of low-, intermediate-, high-risk subsets

Subset (total score)	Score	No. of patients (%)	OS (95% CI)
Low-risk	0	3 (1.5)	
(score 0-2)	1	10 (5.1)	
	2	31 (15.7)	
	Subtotal	44 (22.2)	90.7 (63.7 to 117.6)
Intermediate-risk	3	45 (22.7)	
(score 3-4)	4	55 (27.8)	
	Subtotal	100 (50.5)	48.2 (36.3 to 60.0)
High-risk	5	36 (18.2)	
(score 5-8)	6	16 (8.1)	
	7	1 (0.5)	
	8	1 (0.5)	
	Subtotal	54 (27.3)	40.2 (35.6 to 44.8)

Abbreviation: OS: overall survival; 95% CI: 95% confidence interval.

## Discussion

Unlike other head and neck squamous cell carcinomas, NPC is a highly chemo- and radiosensitive tumor [3]. An intergroup study compared concurrent chemoradiotherapy (CCRT) with radiotherapy alone and found a significant improvement in survival [23-27]. However, the cases of long-term survivors were anecdotal. Most patients succumbed to DM [5,6]. Of the patients with metastases, those with lung metastases comprised a distinct group with a better prognosis and length of survival [8,9,13-15]. Kwan and associates reported that an 18-year-old patient with NPC and intrathoracic metastases survived disease-free for 5 and a half years after primary therapy [28]. Despite the many reports and the literature on prognostic factors and survival rates in patients with NPC [29-34], the present study is novel because the cohorts were limited to a specific site of metastasis(es), the lungs. Based on the unique aetiology, patient characteristics, uniform therapies and long follow-up after the primary treatment, our study demonstrated several clinical factors that are associated not only with LMS but also with OS. Moreover, three risk subsets have been defined, based on the prognostic factors. These subgroups may aid clinicians in selecting the appropriate treatment strategies for patients.

Compared with previous reports, we examined both LMS and OS. We believe that the disease has an integral course that cannot be divided into several parts. Only considered LMS was contrasted to the point that DM originated from occult dissemination at the first diagnosis of NPC and/or at the onset of primary therapies [35]. In addition, the definition of LMS was influenced by the time of diagnosis of DM, which was in turn influenced by the regularity of follow-up. As a consequence, we also examined OS, which is a more informative and appropriate interval. The impact of DFI on LMS and OS was not ignored in our analysis. We found that the use of DFI, LMS and OS as the outcome measures identified the more comprehensive and credible prognostic factors and minimised potential biases.

Consistent with the findings reported by the previous studies, we confirmed that the independently significant negative predictive factors for survival included advanced increased age, T classification, N classification and VCA-IgA titre [3,33,36-38].

Despite some earlier studies that suggested that the number of metastasis(es) and the site of metastasis(es) were not related to survival [13,39,40], we found a statistically significantly different survival rate between patients with solitary and multiple metastases and between unilateral and bilateral pulmonary metastases. The discrepancies between the findings in the literature and our study are likely the result of the different methods that were used to assess the survival outcome in various cohorts of patients. However, this conclusion merits additional research. However, our study failed to demonstrate the correlations between size of lung metastasis(es) and survival by either a univariate or multivariate analysis (*P >*0.05). Furthermore, we investigated the impact of mediastinal node metastases on survival. Regardless of the status of mediastinal lymph nodes, there was no significant difference in survival. Adenopathy was defined by CT imaging as a lymph node > 1 cm in size in the short-axis diameter. We postulated that the use of node size to predict involvement by the tumor had some limitations. For example, some patients with micrometastases may not be detected, and enlarged lymph nodes from other causes may be wrongly diagnosed. Moreover, the various mediastinal node metastases might lead to various prognoses. For example, metastases in mediastinal and/or subcarinal lymph nodes may present more extensive spread than peribronchial and/or hilar and intrapulmonary lymph nodes. Local recurrence has been widely recognised as an independent prognostic factor [9,30]. Notably, local recurrence did not predict survival in our study. Although there was a trend that the patients with lung metastasis(es) that were concurrent with local recurrence had a shorter median OS than patients without local recurrence (46.7 vs. 52.8 months), a statistical difference was not observed between the two groups. This may be due to lack of uniform assessment of local recurrence and histological evidence. Additionally, we cannot detect the micro-recurrence of nasopharynx and regional neck lymph nodes. We have shown that in the current study, for the first time, secondary metastases correlated negatively with survival. Future study should focus on adequate and meticulous collection and analysis of the complaints suggesting micrometastases in the course of managing the NPC patient which may improve the usefulness of this predictive factor. The impact of the DFI on survival has been well documented and discussed. Various investigators chose different cut-off points for the DFI[12,13,41]. In this study, we found a statistically significant correlation between the DFI (≤24 months vs. >24 months) and survival.

In the design of this study, we hoped to identify prognostic factors for lung metastatic NPC patients and to stratify patients into different risk categories. The survival outcomes of the low-, intermediate- and high-risk subsets were significantly different. We thought those subsets would help in a more accurate assessment of a patient's prognosis in the clinical setting and could facilitate the establishment of patient-tailored medical strategies and supports. The outcome of low-risk patients is excellent. The 3-, 5- and 10-year survival rates of the low-risk subset were 77.3%, 60%, and 59%, respectively. We should focus on bringing long-term survival and reducing treatment associated toxicities and complications. Intermediate-risk patients have a modest outcome. The natural history and management of metastatic NPC patients has been long an area of controversy. Our results shown that The 3-, 5- and 10-year survival rates of the intermediate-risk subset were 52.3%, 30%, and 27.8%, respectively. Thus, among those patients, future trials should reevaluate the benefit of sequentially aggressive treatments, such as concurrent chemoradiotherapy and palliative operation. Patients in high-risk subset have poorer prognosis with 3-, 5- and 10-year survival rates as follow: 20.5%, 7%, and 0%. Future studies should focus on relieving clinical symptoms and improving quality of life. We think that these predictive factors and risk groupings could facilitate the establishment of patient-tailored medical strategies and supports.

We acknowledged the limitations of our retrospective analyses. Firstly, not all patients had CT scan of thorax and/or abdomen at the time of diagnosis of NPC, and it is possible that some patients had micrometastasis at the time of diagnosis of NPC which cannot be detected by Chest X-ray and/or ultrasound. Secondly, follow-up CT scan of thorax was not standardized and typically only performed in the patients with abnormal chest X-ray findings. This would, underestimate the true risk of developing lung metastasis(es). If the CT scan of chest and/or PET/CT were used as the standardized follow-up, some micrometastasis in lung missed by X-ray might be detected. However, the clinical and radiographic picture was consistent with lung metastasis(es) from NPC. The primary strength of our study was unique aetiology, patient characteristics, uniform therapies and long follow-up analyzed, which facilitated identifying multiple clinicopathological risk parameters in lung metastatic NPC patients.

## Conclusions

Our study is the first to focus on the prognostic factors and outcomes in NPC patients with pulmonary metastasis(es). We illustrated that age > 45 years, advanced T classification and N classification, elevated VCA-IgA titre, bilateral lung metastases, multiple lung metastases, secondary metastases and a DFI≤24 months were independent, significant and negative factors affecting OS or LMS. The prognosis of the low-, intermediate- and high-risk subsets based on these prognostic factors were significantly different. Thus, we would obtain a more accurate and appropriate assessment of the prognosis of a lung metastatic NPC patient and could facilitate the establishment of patient-tailored medical strategies and supports.

## Competing interests

The authors declare that they have no competing interests.

## Authors' contributions

XC carried out data acquisition, performed the statistical analysis, drafted the manuscript and participated in the sequence alignment. RZL participated in the design of the study and participated in the sequence alignment. YL, LRH and WQL participated in the sequence alignment. YFC carried out data acquisition. ZSW conceived of the study, and participated in its design and coordination and helped to draft the manuscript. All authors read and approved the final manuscript.
